# Determinants for Activation of the Ion Channel TRPV3 by Weak Acids

**DOI:** 10.3390/ijms26178275

**Published:** 2025-08-26

**Authors:** Daniel Rudolf, Inês C. A. Pombeiro Stein, Toni Sturhahn, Julian Wunder, Axel Hage, Andreas Leffler

**Affiliations:** Department of Anesthesiology and Intensive Care Medicine, Hannover Medical School, 30625 Hannover, Germany; rudolf.daniel@mh-hannover.de (D.R.); stein.ines@mh-hannover.de (I.C.A.P.S.); sturhahn.toni@mh-hannover.de (T.S.); wunderjulian@web.de (J.W.); hage.axel@mh-hannover.de (A.H.)

**Keywords:** TRPV3, acidosis, modulation site, weak acid, proton

## Abstract

Several transient receptor potential vanilloid (TRPV) ion channels are proton-sensitive, and recent structural studies have identified poorly conserved mechanisms for the proton sensitivity of TRPV1, TRPV2 and TRPV5. While such detailed studies are lacking for TRPV3, three distinct intracellular motifs were suggested to be required for a direct channel activation by cytosolic acidification. In this study, we investigated if these mechanisms are also relevant for the activation of TRPV3 by weak acids. Wildtype (WT) and several mutants of human TRPV3 transiently expressed in HEK 293T cells were investigated by whole-cell patch clamp electrophysiology. Cells expressing TRPV3-WT generated membrane currents induced by acetic acid (HOAc), formic acid and carbonic acid at pH 5.0. Activation induced by HOAc was concentration-dependent and increased with decreasing pH values. HOAc also strongly potentiated TRPV3-mediated responses to carvacrol and heat. Among the three suggested motifs for the binding of intracellular protons, only the mutant TRPV3-Asp512Ala exhibited an almost complete loss of HOAc sensitivity. The mutation of two C-terminal charged residues (Gln689/Asp727) even resulted in a clear gain of function for both HOAc and heat, and the mutation of the 2-APB-binding site His426 did not significantly abrogate HOAc sensitivity. Finally, insertion of the recently identified binding site in TRPV2 for the weak acid probenecid into TRPV3 (Glu216His) resulted in an increased HOAc sensitivity. To conclude, our data confirm that TRPV3 is sensitized and activated by several weak acids. While Asp512 appears to be a critical intracellular proton-modulating site, a more profound understanding of the mechanisms dictating the proton sensitivity of TRPV3 may require structural studies.

## 1. Introduction

TRPV3 is a nonselective cation channel and a member of the transient receptor potential vanilloid (TRPV) family. TRPV3 is highly expressed in keratinocytes where it plays important roles for cutaneous sensations, hair development and barrier function [1,2]. Inherited gain-of-function mutations in TRPV3 are associated with “Olmsted syndrome”, a rare congenital cutaneous disorder also known as mutilating palmoplantar keratoderma (PPK) [3,4,5]. TRPV3 is also expressed within the central nervous system where it was suggested to be relevant for neuronal damage following brain ischemia [6,7]. For both these and several further conditions, TRPV3 is regarded as an attractive target of novel therapeutics [1,2].

Matsui and colleagues demonstrated that TRPV3 mediates corneoptosis, e.g., cell death of keratinocytes in the upper stratum granulosum of the skin [8]. A strong intracellular acidification was found to be a critical trigger of corneoptosis, but the authors did not take a direct effect of intracellular protons on TRPV3 as a possible mechanism into account [9,10,11]. However, an earlier study on TRPV3 demonstrated that the weak acid glycolic acid gates TRPV3 by diffusing across the cell membrane to induce intracellular acidosis [12]. Protons applied to the cytosolic side indeed activate TRPV3, and the 2-APB binding site His426 was predicted to be a critical intracellular proton modulation site [12,13]. However, the only marginally reduced proton sensitivity of the mutant TRPV3-His426Asp did not fully support that interpretation. Nevertheless, glycolic acid and, further, α-hydroxyl acids support keratinization and are used in skin cosmetics for chemical peeling and anti-aging effects [14,15]. Cao et al. demonstrated that TRPV3 mediates glycolic acid-induced cytotoxicity in cultured keratinocytes, conducting an early in vitro experiment possibly showing the more recently identified concept of corneoptosis [12].

The same laboratory recently demonstrated that inhibition of TRPV3 expressed in cerebral neurons can strongly mitigate neuronal injury following acute ischemia [6]. As the principal mechanism, intracellular acidosis driven by an accumulation of lactate was suggested to activate TRPV3 via His426 [6]. Except for the understanding that protons bind to His426 to gate TRPV3, two additional or rather alternative mechanisms have been proposed, as follows: 1. Gao et al. described that an exchange of any of four residues (Lys508, Asp512, Ser518 and Ala520) in the S2–S3 linker results in a complete insensitivity to cytosolic protons [16]; 2. intracellular protons were suggested to sensitize TRPV3 by protonating C-terminal glutamate and aspartate residues (Glu682, Glu689, Asp727) [17]. The effects of weak acids were not examined in neither of these two studies. Given the proposed physiological and even therapeutic relevance of the weak-acid sensitivity of TRPV3, we aimed to clarify which of the three proposed mechanisms account for weak-acid sensitivity. Hence, we examined the sensitization and activation of TRPV3 induced by weak acids using wildtype as well as several mutant constructs of human TRPV3.

## 2. Results

### 2.1. Weak Acids Sensitize and Activate TRPV3

**Figure 1:**
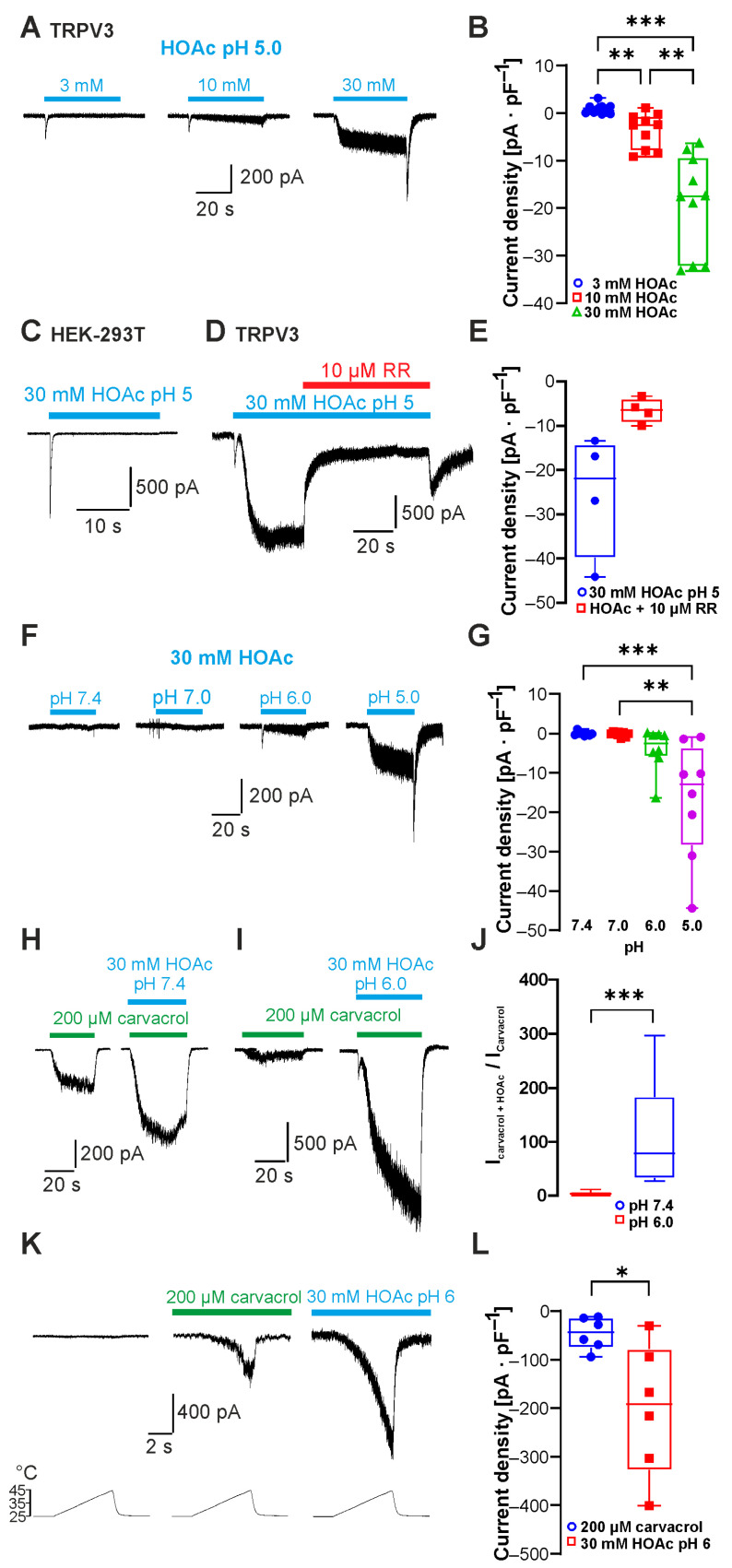
Acetic acid sensitizes and activates TRPV3. (**A**) Inward currents evoked by 3, 10 and 30 mM HOAc at pH 5.0. (**B**) Box plot of respective mean current densities (repeated measures one-way ANOVA and Tukey’s multiple comparisons test). (**C**) Results showing that 30 mM HOAc at pH 5.0 did not activate sustained currents in non-transfected cells. (**D**) Application of 10 µM ruthenium red (RR) inhibited inward currents induced by HOAc in cells expressing TRPV3. (**E**) Box plot of mean densities of currents induced by HOAc without and with 10 µM RR (Wilcoxon matched-pairs signed-rank test). (**F**) Inward currents evoked by 30 mM HOAc at pH 7.4, 7.0, 6.0 and 5.0. (**G**) Box plot of respective mean current densities. (**H**,**I**) Inward currents evoked by 200 µM carvacrol followed by carvacrol co-applied with 30 mM HAOc at pH 7.4 (**H**) and pH 6.0 (**I**). (**J**) Box plot of respective normalized currents (Mann–Whitney U test). (**K**) Heat-evoked inward currents during application of 200 µM carvacrol or 30 mM HOAc at pH 6.0. (**L**) Box plot of respective mean current densities (Wilcoxon matched-pairs signed-rank test). Box plots show medians, from min to max. * *p* < 0.05; ** *p* < 0.01; *** *p* < 0.001.

The undissociated form of weak acids can permeate the cell membrane and induce intracellular acidosis. To date, only glycolic acid has been described to activate TRPV3 by intracellular acidification [12]. To examine the effect of other weak acids on TRPV3 activity, we first utilized acetic acid (HOAc) as a common organic acid with a pK_a_ value of 4.76. In order to examine if HOAc activates TRPV3, HEK 293T cells transiently expressing TRPV3 were examined by means of whole-cell patch clamp. We gradually increased the concentration of HOAc to 3, 10 and 30 mM, and the pH value was titrated to 5.0 at which ~36% of HOAc is membrane-permeable. As is demonstrated in Figure 1A,B, HOAc induced a concentration-dependent activation of inward currents in cells expressing TRPV3 held at −60 mV (n = 10; 3 vs. 10 mM HOAc at pH 5.0, *p* = 0.0075; 10 vs. 30 mM HOAc pH 5.0, *p* = 0.0011; 3 vs. 30 mM HOAc pH 5.0, *p* = 0.0004). In non-transfected HEK 293T cells, 30 mM HOAc at pH 5.0 did not evoke any sustained inward currents but stimulated only rapidly inactivating currents likely to be generated by endogenous acid-sensing ion channels (ASICs, Figure 1C, n = 6). Accordingly, the HOAc-induced currents in TRPV3-expressing cells were strongly inhibited by the unspecific TRPV channel inhibitor ruthenium red (RR 10 µM, 87 ± 5% inhibition, n = 4, Figure 1D,E). These data indicate that TRPV3 accounted for the observed sustained currents induced by HOAc. We next asked if this activation of TRPV3 was pH-dependent. Cells were exposed to 30 mM HOAc, and the pH-value of the external solution was changed from pH 7.4 to 7.0, 6.0 and 5.0 (Figure 1F). The inward currents gradually increased with a significant activation of TRPV3 at pH 5.0 (Figure 1G; n = 8; 30 mM HOAc at pH 5.0 vs. pH 7.0, *p* = 0.0014; 30 mM HOAc at pH 5.0 vs. pH 7.4, *p* = 0.0006). We next aimed to examine if HOAc potentiates channel activation induced by other TRPV3 agonists. 2-APB is a very potent activator of TRPV3, and acidosis was shown to strongly increase the activating potency of 2-APB on TRPV1, TRPV2 and TRPV3 [16]. However, this effect was shown to be primarily due to a direct modification of 2-APB itself by protons and not to the effects of protons on the ion channels [16]. As this proton sensitivity of 2-APB would prohibit valid conclusions in regard to the proton sensitivity of TRPV3, we employed the TRPV3 agonist carvacrol for further investigations. Application of 30 mM HOAc at both pH 7.4 and 6.0 potentiated the inward currents induced by 200 µM carvacrol (Figure 1H,I; n = 9 and 8). This effect was significantly stronger at pH 6.0 compared to pH 7.4 (Figure 1J, *p* = 0.0004). We also examined the effect of both carvacrol and HOAc on heat-induced activation of TRPV3. As is demonstrated in Figure 1K, the combined application of heat and either 200 µM carvacrol or 30 mM HOAc at pH 6.0 resulted in a strong potentiation of heat-evoked currents (Figure 1L; n = 5, *p* = 0.0312).

**Figure 2:**
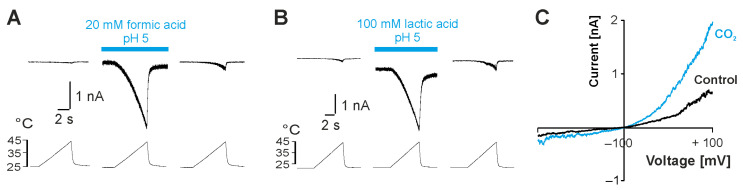
Weak acids modify TRPV3 activity. (**A**,**B**) Heat-evoked inward currents before, during and after application of 20 mM formic acid at pH 5.0 (**A**) or 100 mM lactic acid at pH 5.0 (**B**). (**C**) Membrane currents evoked by 500 ms long voltage ramps from −100 to +100 mV during application of a carbonated solution (CO_2_) at pH 5.0.

We next asked if more physiological weak acids act as endogenous modulators of TRPV3 and chose formic acid, lactic acid and carbon dioxide (CO_2_) for further investigations. After application of either 20 mM formic acid at pH 5.0 (Figure 2A; n = 7) or 100 mM lactic acid at pH 5.0 (Figure 2B, n = 2), the heat-evoked inward currents increased in cells expressing TRPV3. This activation was reversible upon washout. Taken that the focal application of a carbonated solution was hampered due to the loss of gigaseal formation by air bubbles, the solution of the whole dish was replaced. This approach did not produce large inward currents, but when the system was monitored during voltage ramps from −100 to +100 mV, we observed membrane currents with a prominent outward rectification (Figure 2C, n = 2). These data demonstrate that physiologically relevant weak acids can activate TRPV3.

### 2.2. The 2-APB-Binding Site His426 Is Not Required for Sensitivity to Weak Acids

**Figure 3:**
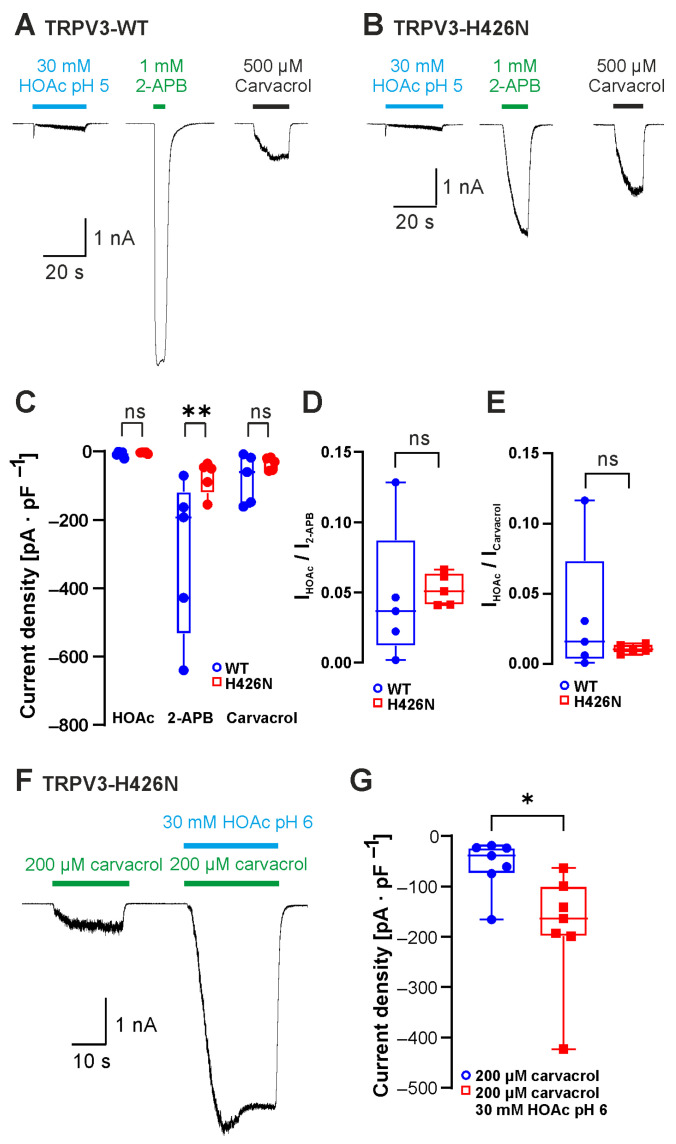
The 2-APB-binding site His426 is not required for sensitivity to weak acids. (**A**,**B**) Inward currents evoked by 30 mM HOAc at pH 5.0, 1 mM 2-APB and 500 µM carvacrol in cells expressing TRPV3-WT (**A**) and TRPV3-H426N (**B**). (**C**) Box plot of the mean current densities of the inward currents induced by HOAc, 2-APB and carvacrol shown in (**A**,**B**) (repeated-measures two-way ANOVA and Tukey’s multiple comparisons test). (**D**,**E**) Box plot of normalized HOAc-induced current densities (Mann–Whitney U test). The peak current amplitudes evoked by HOAc were normalized with currents evoked by 2-APB (**D**) or carvacrol (**E**). (**F**) Inward currents evoked by 200 µM carvacrol followed by carvacrol co-applied with 30 mM HAOc at pH 6.0 in a cell with TRPV3-H426N. (**G**) Box plot of the respective mean current densities (Wilcoxon matched-pairs signed-rank test). Box plots show medians, from min to max. * *p* < 0.05; ** *p* < 0.01, ns denotes not significant.

Having established that weak acids can gate TRPV3, we next examined if the postulated mechanisms for acid sensitivity of TRPV3 are relevant for this effect. The Wang laboratory suggested that the intracellular 2-APB-binding site His426 accounts for weak-acid sensitivity of TRPV3 [6,12,13]. We therefore created the mutant construct TRPV3-His426Asn and examined its activation induced by 30 mM HOAc at pH 5.0, 1 mM 2-APB and 500 µM carvacrol (Figure 3A,B, n = 5 for both genotypes). When comparing the current densities between TRPV3-WT and TRPV3-His426Asn, a significant reduction was evident for activation by 2-APB but not for currents induced by HOAc or carvacrol (Figure 3C, *p* = 0.0019). When we normalized the HOAc-induced current amplitudes with the amplitudes evoked by either 2-APB (Figure 3D) or carvacrol (Figure 3E), we still did not observe any significant differences between TRPV3-WT and TRPV3-His426Asn. Similar to TRPV3-WT, 30 mM HOAc at pH 6.0 also strongly potentiated the carvacrol-induced inward currents in cells expressing TRPV3-His426Asn (Figure 3F,G, n = 7, *p* = 0.0156). Even if these data do not rule out that His426 may be one of several relevant proton modulation sites, this site does not seem to have a prominent role in determining weak-acid sensitivity.

### 2.3. The Carvacrol-Binding Site Asp512 Is Required for Sensitivity to Weak Acids

**Figure 4:**
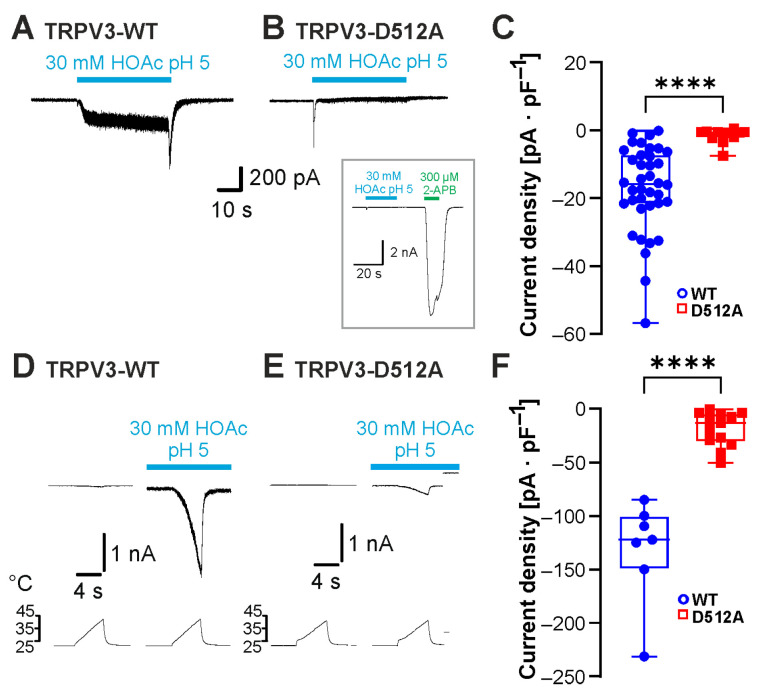
The carvacrol-binding site Asp512 is required for sensitivity to weak acids. (**A**,**B**) Inward currents evoked by 30 mM HOAc at pH 5.0 in cells expressing TRPV3-WT (**A**) and TRPV3-D512A (**B**). (**C**) Box plot of the respective mean current densities (Mann–Whitney U test). (**D**,**E**) Heat-evoked inward currents during the application of 30 mM HOAc at pH 5.0 in cells expressing TRPV3-WT (**D**) or TRPV3-D512A (**E**). (**F**) Box plot of the respective mean current densities (Mann–Whitney U test). Box plots show medians, from min to max. **** *p* < 0.0001.

The four cytoplasmic residues Leu508, Asp512, Ser518 and Ala520 in the S2–3 linker of TRPV3 were demonstrated to be required for activation by intracellular protons [16]. In a more recent study, Leu508 and Asp512 were also identified as likely binding sites for carvacrol [18]. In order to examine the role of this motif in weak-acid sensitivity, the mutant TRPV3-Asp512Ala was examined. HOAc induced inward currents in cells expressing TRPV3-WT (Figure 4A, n = 38) but not in cells expressing TRPV3-Asp512Ala (Figure 4B, n = 11, Figure 4C; *p* < 0.0001). TRPV3-Asp512Ala generated large inward currents in the presence of 300 µM 2-APB, confirming functionality (Figure 4B inset, n = 7). We next asked if the combination of heat and HOAc could activate TRPV3-Asp512Ala. While 30 mM HOAc at pH 5.0 induced a strong but reversible potentiation of heat-evoked inward currents in cells expressing TRPV3-WT (Figure 4D, n = 7), cells expressing TRPV3-Asp512Ala only produced small heat-evoked inward currents in the presence of HOAc (Figure 4E, n = 13, Figure 4F; *p* < 0.0001). The data indeed indicated that weak acids employ the carvacrol-binding site Asp512 to gate TRPV3.

### 2.4. Neutralization of C-Terminal Protonatable Residues Increases Sensitivity to HOAc

**Figure 5:**
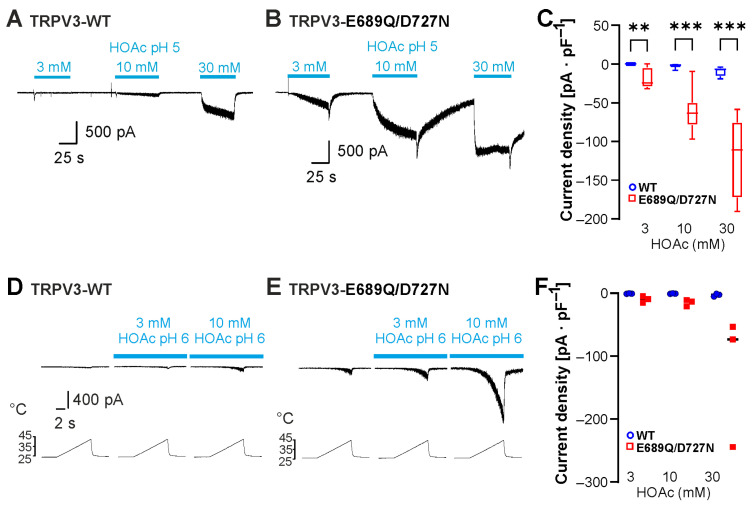
Neutralization of C-terminal protonatable residues increases sensitivity to HOAc. (**A**,**B**) Inward currents generated in cells expressing TRPV3-WT (**A**) or TRPV3-E689Q/D727N (**B**). Cells were exposed to 3, 10 and 30 mM HOAc at pH 5.0. (**C**) Grouped box plots of respective mean current densities (repeated measures two-way ANOVA and Tukey’s multiple comparisons test). (**D**,**E**) Heat-induced inward currents generated in cells expressing TRPV3-WT (**D**) or TRPV3-E689Q/D727N (**E**) before and during application of 3 and 10 mM HOAc at pH 6.0. (**F**) Grouped scatter plots of respective mean current densities. ** *p* < 0.01; *** *p* < 0.001.

The most recent study examining the mechanisms of proton-evoked modulation of TRPV3 suggested that protons induce channel sensitization by interacting with the C-terminal protonatable residues Glu682, Glu689 and Asp727 [17]. The authors demonstrated that a TRPV3-Glu689Gln/Asp727Asn double mutant as well as a TRPV3-Glu682Gln/Glu689Gln/Asp727Asn triple mutant exhibited a reduced potentiation by protons as well as an inhibition rather than an activation by protons in inside-out patches [17]. We examined the effect of HOAc on TRPV3-Glu689Gln/Asp727Asn. Because initial experiments suggested an increased instead of a decreased sensitivity to HOAc, we applied 3, 10 and 30 mM HOAc at pH 5.0. As is demonstrated in Figure 5A,B, the HOAc-induced currents were considerable larger in cells expressing TRPV3-Glu689Gln/Asp727Asn compared to those expressing TRPV3-WT (Figure 5A–C, n = 8; WT vs. E689Q/D727N; 3 mM HOAc, *p* = 0.0038; 10 mM HOAc, *p* = 0.0004; 30 mM HOAc, *p* = 0.0005). Next, we examined the effects of the combination of heat and HOAc on TRPV3-Glu689Gln/Asp727Asn. Because of the expected increase, we restricted HOAc concentration to 3 mM and 10 mM and titrated the pH to 6.0. Again, HOAc induced a potentiation of heat-induced currents that appeared more pronounced for TRPV3-Glu689Gln/Asp727Asn than for TRPV3-WT (Figure 5D,E, n = 3). Because this study aimed at identifying mechanisms mediating proton sensitivity, we did not further investigate the obvious gain-of-function phenotype of TRPV3-Glu689Gln/Asp727Asn. Importantly, neutralization of these C terminal residues did not result in the expected reduction or completely lack of weak-acid sensitivity.

### 2.5. Asp641 Is Relevant for Inhibition of Weak-Acid-Induced Currents by Protons and Calcium

**Figure 6:**
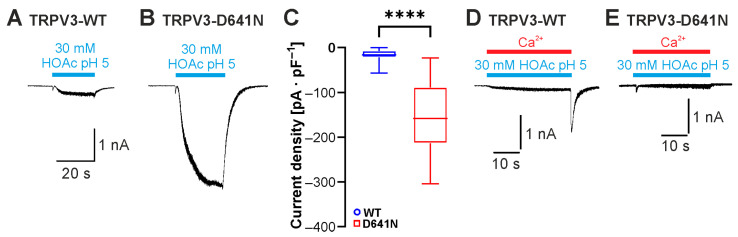
Asp641 dictates inhibition by extracellular protons and calcium. (**A**,**B**) Inward currents generated in cells expressing TRPV3-WT (**A**) or TRPV3- D641N (**B**). Cells were exposed to 30 mM HOAc at pH 5.0. (**C**) Box plot of respective mean current densities (Mann–Whitney U test). (**D**,**E**) Inward currents generated in cells expressing TRPV3-WT (**D**) or TRPV3-D641N (**E**). Cells were exposed to 30 mM HOAc in a solution containing 2 mM Ca^2+^ titrated to pH 5.0. **** *p* < 0.0001.

Another highly relevant effect of extracellular protons on TRPV3 is inhibition [12,17]. Asp641 in the extracellular pore loop was initially identified as a determinant for inhibition by extracellular Ca^2+^ [19]. A subsequent study showed that is also important for inhibition by protons, and extracellular acidification with co-application of 2-APB or carvacrol on the mutant TRPV3-Asp641Asn showed a reduced inhibition by protons [17]. Considering that the inward currents evoked by HOAc at pH 5.0 were generally very small, we asked if this might be due to a simultaneous inhibition by extracellular protons. Indeed, application of 30 mM HOAc at pH 5.0 on cells expressing TRPV3-Asp641Asn evoked significantly larger currents than those observed with TRPV3-WT. (Figure 6A–C; n = 10, *p* < 0.0001). Because of the known inhibition of TRPV3 by intracellular calcium, all experiments in this study were performed in nominally calcium-free solutions. Knowing that both calcium and protons inhibit TRPV3 by interacting with Asp641, we examined the effect of 2 mM calcium on currents induced by 30 mM HOAc at pH 5.0. Indeed, during the application of Ca^2+^, the activation of TRPV3 by HOAc only generated very small currents (Figure 6D; n = 6). Immediately after the removal of the calcium-containing solution with HOAc, we saw a rapidly activating and inactivating inward current that probably reflected a rapid relief of the inhibition. In cells expressing TRPV3-Asp641Asn, however, the small HOAc-evoked currents were not followed by these small “washout currents” (Figure 6E, n = 4). These data suggest that both extracellular calcium and protons regulate the weak-acid-induced activation of TRPV3 by interacting with Asp641.

### 2.6. Insertion of the Protonatable Probenecid-Binding Site from TRPV2 into TRPV3 Increases Weak-Acid Sensitivity

**Figure 7:**
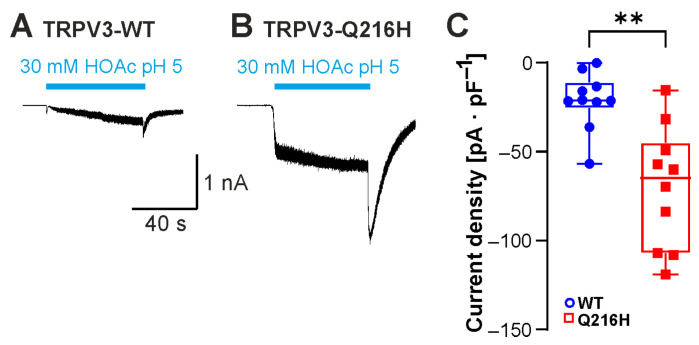
Insertion of the protonatable probenecid-binding site from TRPV2 into TRPV3 increases weak-acid sensitivity. (**A**,**B**) Inward currents generated in cells expressing TRPV3-WT (**A**) or TRPV3- Q216H (**B**). Cells were exposed to 30 mM HOAc at pH 5.0. (**C**) Box plot of respective mean current densities (Mann–Whitney U test). ** *p* < 0.01.

In a recent study, the weak acid probenecid was found to activate TRPV2 by binding the N-terminal His165 [20]. Insertion of a histidine into the corresponding position in TRPV3 (Gln216His) resulted in de novo probenecid sensitivity of TRPV3 [20]. We finally asked if this additional protonatable residue in TRPV3 might also produce a gain-of-function phenotype in response to HOAc. Compared to TRPV3-WT, TRPV3-Gln216His indeed generated significantly larger HOAc-evoked currents (Figure 7A–C; n = 10, *p* = 0.0015).

## 3. Discussion

**Figure 8:**
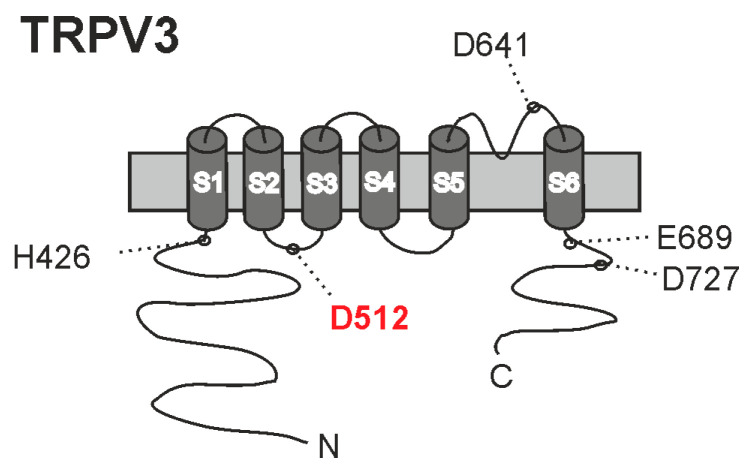
Cartoon of TRPV3 with the putative proton-binding sites suggested in previous studies. In this study, we found thatD512 is the principal proton-binding site for activation by weak acids.

In this in vitro study we examined the effects of weak acids on TRPV3. The primary aim was to determine the molecular mechanism for weak-acid sensitivity, and our strategy to achieve this was to re-examine three mechanisms that were previously suggested to account for the sensitivity of TRPV3 to intracellular protons. Although it seemed likely that the sensitivity to weak acids would be dictated by the same mechanisms as the sensitivity to intracellular protons, this is the first study addressing this question. Our data indeed support the understanding that weak acids activate TRPV3 by diffusing through the cell membrane to induce intracellular acidosis. Among the three examined intracellular mechanisms, only one seems to be relevant for weak-acid sensitivity (Figure 8). Replacement of the carvacrol-binding site Asp512 resulted in a more or less complete loss in weak-acid sensitivity, but with a retained channel function. Thus, carvacrol and weak acids seem to employ a common intracellular mechanism to activate TRPV3, and we suggest that the carvacrol-binding site Asp512 can be an important modulation site of TRPV3 in terms of structure-based development of selective TRPV3 modulators. This site is not conserved in the proton-sensitive TRPV channels TRPV1 or TRPV2, thus suggesting that distinct mechanisms account for the proton sensitivities of these closely related ion channels. As for TRPV2 and TRPV3, only few studies have yet studied their proton sensitivities.

TRPV3 is activated by several synthetic chemicals including 2-APB but also by natural compounds including monoterpenes and cannabinoids [1]. The molecular pharmacology of several of these agonists, but also of antagonists, has been studied at the structural level in reports combining functional assays with cryo-electron microscopy (cryo-EM) [21,22,23,24,25,26,27]. This approach has revealed the exact binding sites for 2-APB, monoterpenes and tetrahydrocannabivarin, as well as for antagonists like dyclonine and osthole [21,22,23,24,25,26,27]. With this richness of both structural and functional data on the molecular pharmacology of TRPV3, it even seems somewhat surprising that the mechanisms of action of protons have yet to be explored at the structural level. Eventually, the relatively few published reports on the proton sensitivity of TRPV3 might indicate that this property is not yet regarded as relevant enough to invest the required resources in its study.

The closely related capsaicin receptor TRPV1 is activated by extracellular protons [28,29], a property that has been intensively studied due to its likely relevance for inflammatory pain. The initial identification of the extracellular proton-binding sites of TRPV1 was achieved by a mutational screening approach [28], and a recent cryo-EM study verified this finding [30]. In a study combining electrophysiology and cryo-EM, we recently described the molecular mechanism of activation of TRPV2 by weak acids [31]. In TRPV2, it is evident that weak acids and 2-APB have a common intracellular protonatable binding site (His521 in rat TRPV2 [31]). From this perspective, the idea that intracellular protons bind to the 2-APB-binding site His426 to activate TRPV3 seems plausible [12]. For 2-APB, the original report on His426 as a relevant binding site was confirmed in a cryo-EM-determined structure [13]. For protons, however, the original report from Cao et al. showed that TRPV3-His426Asn generates substantial proton-evoked membrane currents [12]. Accordingly, our data clearly demonstrate that TRPV3-His426Asn is also sensitized and activated by weak acids. These observations allow us to conclude that His426 is obviously not required for the acid sensitivity of TRPV3. However, the experimental setups of both studies would not allow for the detection of a marginal reduction in acid sensitivity; so, it is possible that the proton-induced modulation of TRPV3 function also involves His426 to some extent. This notion is supported by our observation that the replacement of Gln216 with a histidine resulted in a markedly increased sensitivity to HOAc. Gln216 is the corresponding site to His165 in rat TRPV2 that was recently identified as the main binding site for the weak acid probenecid, and the mutant TRPV3-Gln216His, but not TRPV3-WT, exhibited probenecid sensitivity [20]. Thus, several intracellular protonatable residues of TRPV3 may regulate the sensitivity to protons. Along these lines, the suggested role of the C-terminal glutamate and aspartate residues as relevant proton modulation sites in TRPV3 seems justified [17]. In an elaborated study utilizing molecular dynamic simulations and electrophysiology on mutant TRPV3 constructs, Wang et al. concluded that intracellular protons can sensitize TRPV3 by neutralizing the negatively charged residues Glu682, Glu689 and Asp727 [17]. Neutralization of Glu689 and Asp727 or all three residues by mutagenesis stabilized the sensitized conformation of TRPV3, resulting in a gain-of-function phenotype that should not be further sensitized by acidosis. Our data on TRPV3-Glu689Gln/Asp727Asn support a general gain-of-function phenotype, as also heat-induced currents appeared to be increased, but this also applied for sensitivity to weak acids. Thus, activation of TRPV3-Glu689Gln/Asp727Asn by HOAc was not only preserved, but even strongly enhanced. Therefore, it seems legitimate to conclude that at least Glu689 and Asp727 are not required for sensitivity to intracellular acidosis induced by weak acids. Wang et al. also demonstrated that the pore residue Asp641 seems to be an important extracellular site allowing protons to inhibit TRPV3 [17]. Our data on TRPV3-Asp641Asn strongly support this notion, as the mutant produced enhanced the inward currents evoked by HOAc at pH 5.0. Interestingly, this same residue is relevant for the inhibition of TRPV3 by extracellular calcium [12].

In our hands, the only mutant in this study with a clear loss-of-function phenotype in regard to weak-acid-induced sensitization and activation was TRPV3-Asp512Ala. This mechanism for proton sensitivity was identified by Gao et al. who described that the cytoplasmic residues Lys508, Asp512, Ser518 and Ala520 in the S2–S3 linker are required for activation by intracellular protons [16]. This motif was identified by an unbiased approach investigating multiple chimeric TRPV1/TRPV3 constructs, and each of the single mutants with the replacement of any one residue among Lys508, Asp512, Ser518 and Ala520 resulted in a strongly reduced sensitivity to cytosolic protons, with the most prominent effect for Asp512 [16]. We only investigated the protonatable residue Asp512 in our study, but it seems likely that also the replacement of Lys508, Ser518 and Ala520 would result in a loss of weak-acid sensitivity. A more recent study from Niu et al. utilized molecular docking from the published structure of TRPV3 to identify a unique binding pocket in the S2–S3 linker for carvacrol, formed by Iso505, Leu508, Arg509 and Asp512 [18]. While the heat-induced activation of TRPV3 seems to involve conformational changes in several domains of the channel, it was suggested that heat-induced changes in the secondary structure of the S2–S3 linker are important for thermal sensitivity [26]. Accordingly, we demonstrated that weak acids strongly sensitize TRPV3 to activation by both carvacrol and heat. It is important to note that our study remains descriptive and is limited to the functional properties of the human orthologue of TRPV3 heterologously expressed in HEK 293T cells. Further, more elaborated studies are warranted to obtain a more detailed understanding of the mechanisms and implications of this weak-acid sensitivity of TRPV3. These include structural approaches using the purified TRPV3 protein along with molecular dynamic simulations that would allow for a definite identification of relevant proton modulation sites in TRPV3. Nevertheless, we can conclude that our functional data demonstrate that TRPV3 can be sensitized and activated by weak acids and that the main molecular mechanism for this property is likely to be a proton-induced conformational change of a motif in the S2–S3 loop known to dictate TRPV3 sensitivity to both carvacrol and heat.

## 4. Materials and Methods

### 4.1. Chemicals

Chemicals were purchased and dissolved as follows: carvacrol was obtained from Sigma-Aldrich (Taufkirchen, Germany), stored at 4 °C and diluted directly before application; formic acid and lactic acid were purchased from Sigma-Aldrich (Taufkirchen, Germany); and acetic acid, hydrochloric acid, sodium hydroxide, potassium hydroxide, EGTA (ethyleneglycol-bis(β-aminoethyl)-N,N,N′,N′-tetraacetic acid) and MES (s-(N-morpholino)ethanesulfonic acid) were purchased from Merck (Darmstadt, Germany).

### 4.2. Cell Culture

HEK293T cells were transfected with plasmids coding for human TRPV3 and EGFP using the jetPEI^®^ transfection reagent as recently described [20,31] (Polyplus-transfection, Illkirch, France). The cells were cultured in Dulbecco’s modified Eagle medium nutrient mixture F-12 (Gibco^®^ DMEM/F12, Thermo Fisher Scientific, Karlsruhe, Germany) supplemented with 10% fetal bovine serum (Biochrom, Berlin, Germany) at 37 °C in 5% CO_2_. Approximately 24 h after transfection, the cells were detached using phosphate -buffered saline (PBS, Lonza, Cologne, Germany).

### 4.3. Mutagenesis

Wildtype human TRPV3 (plasmid: pcDNA3.1(+)) was a gift from Prof. Peter Zygmunt (Lund, Sweden). TRPV3 mutations were made by using the QuikChange Lightning site-directed mutagenesis kit (Agilent Technologies, Waldbronn, Germany). All mutants were verified by DNA sequencing. Asp641Asn (primers: forward GGATGTTCAGGTTACCCAGGCCTATGGTGAG, reverse CTCACCATAGGCCTGGGTAACCTGAACATCC); Asp512Ala (primers: forward GGATGGACTGCAGAGCCGAGGGTCTAGF, reverse GCTGAGACCCTCGGCTCTGCAGTCCATCC); Glu689Gln(primers: forward GGCGCCAGATGCGCTGGCTCTCCTTGGAG, reverse CTCCAAGGAGAGCCAGCGCATCTGGCGCC)/Asp727Asn (primers: forward CAAACACAGTCGGAAATTATCCTCGGCCACTTTGC, reverse GCAAAGTGGCCGAGGATAATTTCCGACTGTGTTTG); His426Asn (primers: forward GCAGCAGCGTGTTCAGCGGCTCCAG, reverse CTGGAGCCGCTGAACACGCTGCTGC); Gln216His (primers: forward CAGCGCCGTATGCCCTTCATAGGCCTC, reverse GAGGCCTATGAAGGGCATACGGCGCTG).

### 4.4. Patch Clamp

Whole-cell voltage-clamp recordings were performed on HEK293T cells expressing TRPV3. The standard extracellular solution contained the following components in mM concentrations: 140 NaCl, 5 KCl, 2 MgCl_2_, 10 Glucose, 10 HEPES, and 5 EGTA at pH 7.4 adjusted by sodium hydroxide. The extracellular solution with calcium contained the following components in mM concentrations: 140 NaCl, 5 KCl, 2 MgCl_2_, 2 CaCl_2_, 10 Glucose, and 10 MES at pH 7.4 adjusted by sodium hydroxide. The pipette solution contained in mM concentrations: 140 KCl, 2 MgCl_2_, 10 MES, and 5 EGTA at pH 7.4 adjusted by potassium hydroxide. The solutions were bath-applied using a gravity-driven polytetrafluoroethylene/glass multibarrel perfusion system. All recordings were executed at room temperature. The patch pipettes were pulled from borosilicate glass tubes (TW105F-3, World Precision Instruments, Berlin, Germany) and heat-polished to give a resistance of 2–5.0 MΩ. Only cells with an initial seal < 1GOhm and access resistance < 10 MOhm were selected for recordings. The capacitance of the cells was determined with the circuitry of the Patchmaster software (Version 2x92, HEKA Elektronik), and the HEK 293T cells used in this study exhibited a capacitance between 10 and 30 pF. An EPC 9 HEKA amplifier (HEKA Elektronik, Lambrecht, Germany) was used, and the data were sampled at 2 kHz. The cells were held at −60 mV. Heat ramps were performed by using an insulated copper wire around the output connection of the perfusion system passing a current to heat the solution from room temperature to 45–50 °C within 10 s, as described previously [32]. The temperature of the surrounded solution was measured by a miniature thermocouple fixed at the tip of the capillary tube. Data acquisition was performed with Patchmaster software (Version 2x92, HEKA Elektronik), and the data analyses were performed with Fitmaster software (Version v2x92, HEKA Elektronik) and Origin 8.5.1 (Origin Lap, Northampton, MA, USA).

### 4.5. Carbon Dioxide Measurement

Transiently transfected HEK293T cells were exposed to an extracellular solution carbonated with a conventional soda streamer. The pH value was set at pH 5.0.

### 4.6. Statistical Analysis

All recorded data were analyzed using MS Excel (Excel 2010; Microsoft, Seattle, DC, USA), Origin 8.5.1. (Origin Lap, Northampton, MA, USA) and GraphPad Prism (Prism 10; Graph Pad Software Inc., San Diego, CA, USA) and are presented as mean ± standard deviation. For Patch Clamp recordings, only one cell per dish was used. The given n represents the total recorded number of included cells. Normal distribution was tested using the Kolmogorov–Smirnov test and Shapiro–Wilk test. Student’s t-test for normally distributed variables and Mann–Whitney U test for non-parametric variables were used as appropriate to compare between-group differences. For comparisons of >2 groups, one-way analysis of variance (ANOVA) or Kruskal–Wallis test for non-parametric variables as used. For comparisons of >2 groups with two factors for non-parametric variables, we used the two-way ANOVA and Tukey’s multiple comparisons test. For comparisons of 2 paired groups, the Wilcoxon matched-pairs signed-rank test was used for non-parametric variables. For comparisons of >2 paired groups, Friedman’s test was used for non-parametric variables. The level of statistical significance was set at *p* < 0.05.

## Data Availability

All data are presented in the manuscript. Original data can be ob-tained from the corresponding author upon reasonable request.

## Abbreviations

The following abbreviations are used in this manuscript:

TRPV3	transient receptor potential vanilloid 3
HEK293T	human embryonic kidney cells
2-APB	2-aminoethoxydiphenyl borate
HOAc	acetic acid
WT	wildtype
CO_2_	carbon dioxide
FPP	farnesyl pyrophosphate
cryo-EM	cryo-electron microscopy
